# Supplementary material for: Discovery and structural characterization of monkeypox virus methyltransferase VP39 inhibitors reveal similarities to SARS-CoV-2 nsp14 methyltransferase
Source: Nat Commun. 2023 Apr 20;14:2259. doi: 10.1038/s41467-023-38019-1 (PMC10116469; doi:10.1038/s41467-023-38019-1)
Supplement: Supplementary file 1 — Supplementary Information [file 41467_2023_38019_MOESM1_ESM.pdf]

Supplementary Information

Discovery and structural characterization of monkeypox virus methyltransferase VP39 inhibitors reveal similarities to SARS-CoV-2 nsp14 methyltransferase

Jan Silhan, Martin Klima, Tomas Otava, Petr Skvara *et al.*

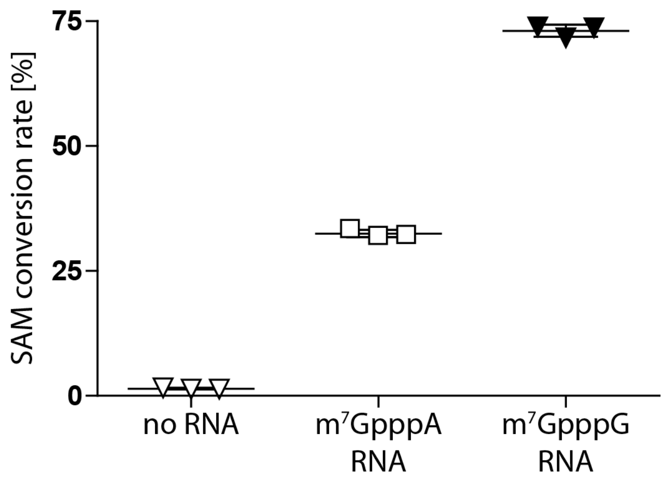

Supplementary Figure 1 Methyltransferase activity of the VP39 protein with capped RNA substrates used as indicated. Data points are presented as mean values ± standard deviations (n = 3). Source data are provided as a Source Data file.

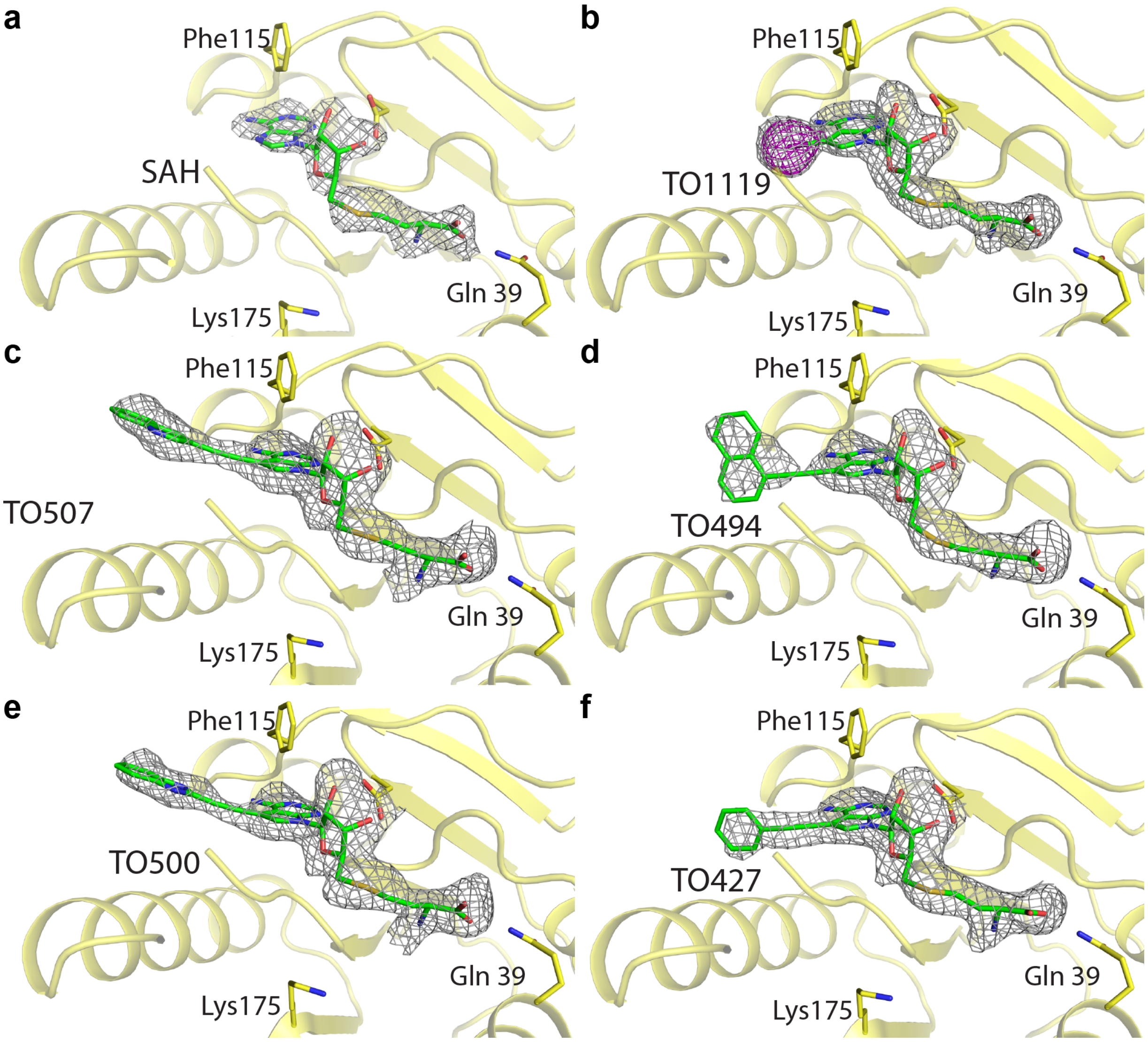

Supplementary Figure 2 Small molecule inhibitors of VP39 methyltransferase and their fits in Fo-Fc maps.

Detail of VP39 protein in complex with SAH and small molecule inhibitors. The Fo-Fcomit map is contoured around the ligand at 2σ (gray mesh). The proximity of the iodine atom of molecule TO1119 is highlighted by the Fo-Fc map contoured at 5σ (purple mesh). The colors and arrangement of the ligands are identical to Figure 3 of the main text.

Supplementary Note 1. Comparison to other viral MTases

Despite the overall similarities of the SAM binding site, differences do exist in the binding mode of the nucleobase as well as the sugar ring. In the case of mpox VP39, the adenine base is bound to the backbone via Val116 (Figure 2) and held in place by a complex water network (Figure 3A), while in SARS-CoV-2 it is held by the side chain of Asp114 (Supplementary Figure 3B) and in Zika by the side chain of Asp131 (Figure 3C). The binding mode of the ribose ring is virtually identical between VP39 and SARS-CoV-2 nsp16, as both of these viruses utilize an aspartate residue (Supplementary Figure 3). However, in Zika it is held in place by His110 and Glu111 (SI Figure 3C). Interestingly, all these viruses use an aspartate residue to interact with the amino group of sinefungin's amino acid moiety, further highlighting the similarity of the SAM binding site between a dsDNA virus and two distinct +RNA viruses.

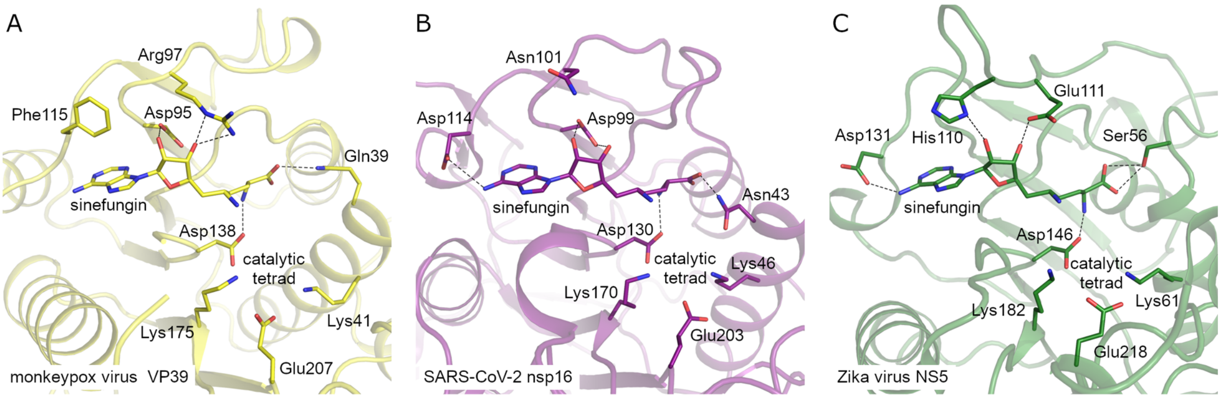

Supplementary Figure 3 Structural alignment of the methyltransferase active sites of monkeypox virus VP39 with SARS-CoV-2 nsp16 and Zika virus NS5. Crystal structures of SARS-CoV-2 nsp10/nsp16 (**a**, pdb entry 6YZ1) and Zika virus NS5 methyltransferase domain (**b**, pdb entry 5MRK) were used for the alignment. Protein backbones are shown in the cartoon representation and depicted in yellow (monkeypox virus VP39), magenta (SARS-CoV-2 nsp16), and green (Zika virus NS5). Sinefungin and the side chains of selected residues are shown in the stick representation with carbon atoms colored according to the protein assignment and other elements colored as in Fig 1B.

Supplementary Methods

General information

Preparation of starting material **1** and synthesis of methyltransferase inhibitors **TO427, TO494, TO500, TO504** and **TO507** were performed according to published procedures.[1] Reagents were purchased from Fluorochem and Sigma Aldrich. Flash column chromatography (normal – VWR International Silica gel 60 from, particle size 40–63 µm or reverse-phase – C18 RediSep Rf columns) were performed using Combiflash® Rf from Teledyne ISCO. Waters UPLC H-Class Core System (column Waters Acquity UPLC BEH C18 1.7 µm, 2.1 mm × 100 mm), Waters Acquity UPLC PDA detector, mass spectrometer Waters SQ D2, and MassLynx mass spectrometry software were used for UPLC analysis of the reaction mixtures. NMR spectra were recorded on a Bruker Avance III^{TM} HD 400 instrument (400.0 MHz for ^{1}H and 101 MHz for ^{13}C) using inverse broadband probe with ATM module (5 mm BBO-^{1}H Z-GRD) or Bruker Avance III^{TM} HD 400 instrument with broadband PRODIGY cryoprobe with ATM module (5 mm CPBBO BB-^{1}H/^{19}F/D Z-GRD). Chemical shifts (δ) and coupling constants (*J*) are expressed in ppm and Hz, respectively. The NMR experiments were performed in DMSO-*d6* and referenced to the solvent signal (δ 2.50 for ^{1}H NMR and 39.70 for ^{13}C NMR). All structures were confirmed and ^{1}H and ^{13}C signals were assigned by a combination of 2D NMR (H,H-COSY, H,C-HSQC, and H,C-HMBC) experiments. The numbering of structures was inspired by numbering of nucleosides (normal digits for nucleobase – 7-deazaadenine, prime digits for carbohydrate moiety), and for amino acid moiety, symbols from Greek alphabet (α, β, γ) were used. High resolution mass spectrometry (HRMS) analyses were carried out on an LTQ XL Orbitrap XL (Thermo Fisher Scientific) using electrospray ionization (ESI). Purity of all prepared compounds was higher than 95%.

Synthetic procedures

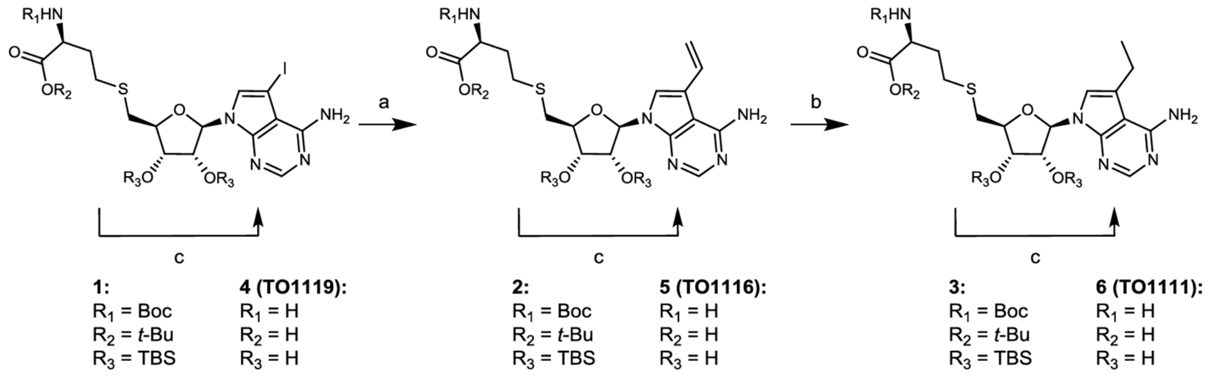

Reagents and conditions: (a) $Pd(PPh_3)_4$, tributylvinylstannane, DMF, 100 °C. (b) H_2-Pd/C, EtOAc/EtOH, r.t. (c) TFA/H_2O (9:1).

***tert*-Butyl *S*-(((2*S*,3*R*,4*R*,5*R*)-5-(4-amino-5-vinyl-7*H*-pyrrolo[2,3-*d*]pyrimidin-7-yl)-3,4-bis((*tert*-butyldimethylsilyl)oxy)tetrahydrofuran-2-yl)methyl)-*N*-(*tert*-butoxycarbonyl)-L-homocysteinate (2):**

An argon atmosphere was introduced to the solution of compound **1** (200 mg, 0.22 mmol), $Pd(PPh_3)_4$ (28 mg, 0.02 mmol) and tributylvinylstannane (260 µL, 0.89 mmol) in dry DMF (4 mL) and the reaction mixture was stirred at 100 °C for 2 h. The solvent was removed under the reduced pressure, the residue was dissolved in DCM (100 mL) and washed with water (50 mL). Organic layer was dried over anhydrous Na_2SO_4, and the solvent was removed in vacuo. Flash column chromatography on silica gel (10 to 70 % EtOAc in DCM) gave protected SAH analogue **2** (147 mg, 0.19 mmol, 83 %) as white foam. **^{1}H NMR** (400 MHz, DMSO-*d*$_6$) δ 8.05 (1H, s, H2), 7.65 (1H, s, H8), 7.17 (1H, d, *J* = 7.9 Hz, NH^{Boc}), 7.11 (1H, dd, *J* = 17.2, 11.0 Hz, CH_2=**CH**), 6.70 (2H, br s, NH_2), 6.13 (1H, d, *J* = 7.3 Hz, H1′), 5.56 (1H, dd, *J* = 17.4, 1.2 Hz, **CH_2**=CH_{trans}), 5.13 (1H, dd, *J* = 12.1, 1.2 Hz, **CH_2**=CH_{cis}), 4.79 (1H, dd, *J* = 7.2, 4.3 Hz, H2′), 4.19 (1H, d, *J* = 4.2, Hz, H3′), 4.01–3.87 (2H, m, Hα, H4′), 3.02 (1H, dd, *J* = 13.7, 8.3 Hz, H5′a), 2.84 (1H, dd, *J* = 13.8, 5.8 Hz, H5′b), 2.65–2.52 (2H, m, Hγ), 1.91–1.75 (2H, m, Hβ), 1.37 (18H, 2×s, *t*Bu^{Boc}, *t*Bu), 0.93, 0.66 (18H, 2×s, 2×*t*Bu^{TBS}), 0.16, 0.12, −0.11, −0.39 (12H, 4×s, 4×Me^{TBS}). **^{13}C NMR** (101 MHz, DMSO-*d*$_6$) δ 171.6 (**CO**O*t*Bu), 157.8 (C6), 155.8 (**CO**O*t*Bu^{Boc}), 151.9 (C2), 151.6 (C4), 129.2 (CH_2=**CH**), 119.0 (C8), 114.9 (C7), 113.4 (**CH_2**=CH), 100.9 (C5), 86.1 (C1′), 84.5 (C4′), 80.5, 78.3 (*t*Buq^{Boc}, *t*Buq), 74.7 (C3′), 74.0 (C2′), 53.5 (Cα), 33.7 (C5′), 31.2 (Cβ), 28.39 (Cγ), 28.35, 27.8 (*t*Bu^{Boc}, *t*Bu), 26.0, 25.7 (2×*t*Bu^{TBS}), 18.0, 17.7 (2×*t*Buq^{TBS}), −4.4, −4.6, −5.4 (4×Me^{TBS}). **HRMS** (ESI) $[M + H]^+$ m/z calcd. for $C_{38}H_{70}O_7N_5SSi_2$, 796.4529; found, 796.4524.

***tert*-Butyl *S*-(((2*S*,3*R*,4*R*,5*R*)-5-(4-amino-5-ethyl-7*H*-pyrrolo[2,3-*d*]pyrimidin-7-yl)-3,4-bis((*tert*-butyldimethylsilyl)oxy)tetrahydrofuran-2-yl)methyl)-*N* -(*tert*-butoxycarbonyl)-L-homocysteinate (3):**

10% Pd/C (36 mg, 0.03 mmol) was added to the solution of 7-vinyl analogue **2** (120 mg, 0.15 mmol) in a mixture of EtOH and EtOAc (5 mL per mmol, 1:1) and H_2 atmosphere was introduced using Schlenk technique. After stirring for 12 h at r.t., the reaction mixture was filtered through celite, and the solvent was removed under reduced pressure. The crude mixture was purified by reverse phase flash column chromatography (25 to 100 % of ACN in H_2O) providing desired SAH analogue **3** (98 mg, 0.12 mmol, 82 %) as a white foam. **^{1}H NMR** (400 MHz, DMSO-*d*$_6$) δ 8.02 (1H, s, H2), 7.17 (1H, d, *J* = 7.9 Hz, NH^{Boc}), 7.09 (1H, s, H8), 6.52 (2H, br s, NH_2), 6.10 (1H, d, *J* = 7.4 Hz, H1′), 4.72 (1H, dd, *J* = 7.3, 4.4 Hz, H2′), 4.17 (1H, d, *J* = 4.2 Hz, H3′), 3.98−3.88 (2H, m, H4′, Hα), 2.98 (1H, dd, *J* = 13.7, 8.1 Hz, H5′a), 2.85–2.73 (3H, m, CH_3**<u>CH_2</u>**, H5′b), 2.66−2.51 (2H, m, Hγ), 1.91−1.76 (2H, m, Hβ), 1.38, 1.37 (18H, 2×s, *t*Bu^{Boc}, *t*Bu), 1.18 (3H, t, *J* = 7.4 Hz, **<u>CH_3</u>**CH_2), 0.93, 0.66 (18H, 2×s, 2×*t*Bu^{TBS}), 0.15, 0.12, −0.12, −0.39 (12H, 4×s, 4×Me^{TBS}). **^{13}C NMR** (101 MHz, DMSO-*d*$_6$) δ 171.6 (**<u>CO</u>**O*t*Bu), 157.7 (C6), 155.8 (**<u>CO</u>**O*t*Bu^{Boc}), 151.7 (C4), 151.4 (C2), 118.2 (C8), 117.7 (C7), 102.2 (C5), 85.8 (C1′), 84.3 (C4′), 80.5, 78.3 (*t*Buq^{Boc}, *t*Buq), 74.7 (C3′), 74.0 (C2′), 53.5 (Cα), 33.8 (C5′), 31.2 (Cβ), 28.5 (Cγ), 28.3, 27.8 (*t*Bu^{Boc}, *t*Bu), 26.0, 25.7 (2×*t*Bu^{TBS}), 19.4 (CH_3**<u>CH_2</u>**), 18.0, 17.7 (2×*t*Buq^{TBS}), 15.3 (**<u>CH_3</u>**CH_2), −4.4, −4.5, −5.4 (4×Me^{TBS}). **HRMS** (ESI) $[M + H]^+$ m/z calcd. for $C_{38}H_{68}O_7N_5SSi_2$; 794.4373; found, 794.4369.

General procedure A: Protecting group removal under acidic conditions. Protected nucleoside analogue was dissolved in a mixture of TFA and water (9:1, 15 mL per mmol) and the reaction mixture was stirred for 3 h at r.t. After completion of the reaction, the volatiles were evaporated under reduced pressure and the residue was co-evaporated twice with MeOH. The crude product was purified by reverse-phase flash column chromatography to provide desired final compounds.

***S*-(((2*S*,3*S*,4*R*,5*R*)-5-(4-Amino-5-iodo-7*H*-pyrrolo[2,3-*d*]pyrimidin-7-yl)-3,4-dihydroxytetrahydrofuran-2-yl)methyl)-L-homocysteine (4, TO1119):**

Derivative **4** was synthesized from compound **1** (100 mg, 0.11 mmol) according to the **general procedure A**. Reverse-phase flash column chromatography (0 to 50 % of ACN in H_2O) gave final compound **4** (49 mg, 0.10 mmol, 87 %) as a white powder. **^{1}H NMR** (400 MHz, DMSO-*d*$_6$) δ 8.11 (1H, s, H2), 7.66 (1H, s, H8), 6.68 (2H, br s, 6-NH_2), 6.05 (1H, d, *J* = 6.2 Hz, H1′), 5.73–5.38 (2H, m, α-NH_2), 4.45 (1H, t, *J* = 5.6 Hz, H2′), 3.96 (1H, dd, *J* = 5.6, 3.5 Hz, H3′), 3.99–3.92 (1H, m, H4′), 3.30 (1H, t, *J* = 6.0 Hz, Hα), 2.88 (1H, dd, *J* = 13.6, 6.4 Hz, H5′a), 2.74 (1H, dd, *J* = 13.6, 6.5 Hz, H5′b), 2.63 (2H, t, *J* =

7.6 Hz, Hγ), 2.07−1.93 (1H, m, Hβa), 1.90−1.76 (1H, m, Hβb). ^{13}**C NMR** (101 MHz, DMSO-d_6) δ 169.8 (**CO**OH), 157.4 (C6), 152.3 (C2), 150.7 (C4), 127.1 (C8), 103.3 (C5), 86.6 (C1′), 83.2 (C4′), 73.2 (C2′), 72.7 (C3′), 53.1 (Cα), 52.6 (C7), 34.1 (C5′), 31.5 (Cβ), 28.3 (Cγ). **HRMS** (ESI): $[M + H]^+$ m/z calcd. for $C_{15}H_{21}O_5N_5IS$, 510.0303; found, 510.0299.

***S*-(((2*S*,3*S*,4*R*,5*R*)-5-(4-Amino-5-vinyl-7*H*-pyrrolo[2,3-*d*]pyrimidin-7-yl)-3,4-dihydroxytetrahydrofuran-2-yl)methyl)-L-homocysteine (5, TO1116):**

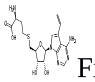

Final compound **5** was prepared from protected SAH analogue **2** (80 mg, 0.10 mmol) using the **general procedure A**. Purification by reverse-phase flash column chromatography (0 to 50 % of ACN with 0.5 % of HCOOH in H_2O with 0.5 % of HCOOH) afforded formate salt of the desired product **5** (28 mg, 0.07 mmol, 68 %) as a white powder. ^{1}**H NMR** (400 MHz, DMSO-d_6) δ 8.22 (1H, s **H**COO$^-$), 8.06 (1H, s, H2), 7.65 (1H, s, H8), 7.11 (1H, dd, J = 17.2, 10.9 Hz, CH_2=**CH**), 6.71 (2H, br s, 6-NH_2), 6.09 (1H, d, J = 6.0 Hz, H1′), 5.61 (1H, dd, J = 17.3, 1.3 Hz, **CH_2**=CH_{trans}), 5.13 (1H, dd, J = 11.0, 1.3 Hz, **CH_2**=CH_{cis}), 4.46 (1H, t, J = 5.6 Hz, H2′), 4.08–4.04 (1H, m, H3′), 4.00–3.92 (1H, m, H4′), 3.33–3.27 (1H, m, Hα), 2.90 (1H, dd, J = 13.6, 6.2 Hz, H5′a), 2.75 (1H, dd, J = 13.4, 6.4 Hz, H5′b), 2.69–2.58 (2H, m, Hγ), 2.05−1.91 (1H, m, Hβa), 1.87−1.70 (1H, m, Hβb). ^{13}**C NMR** (101 MHz, DMSO-d_6) δ 170.5 (**CO**OH), 163.7 (H**C**OO$^-$) 157.8 (C6), 151.9 (C2), 151.4 (C4), 129.2 (CH_2=**CH**), 118.8 (C8), 114.7 (C7), 113.5 (**CH_2**=CH), 100.8 (C5), 86.6 (C1′), 83.0 (C4′), 73.3 (C2′), 72.8 (C3′), 53.0 (Cα), 34.2 (C5′), 31.4 (Cβ), 28.2 (Cγ). **HRMS** (ESI) $[M + H]^+$ m/z calcd. for $C_{17}H_{24}O_5N_5S$, 410.1493; found, 410.1490.

***S*-(((2*S*,3*S*,4*R*,5*R*)-5-(4-Amino-5-ethyl-7*H*-pyrrolo[2,3-*d*]pyrimidin-7-yl)-3,4-dihydroxytetrahydrofuran-2-yl)methyl)-L-homocysteine (6, TO1111):**

Compound **6** was synthesized from derivative **3** (70 mg, 0.09 mmol) according to the **general procedure A**. Reverse-phase flash column chromatography (0 to 50 % of ACN in H_2O) gave desired product **6** (31 mg, 0.08 mmol, 84 %) as a white powder. ^{1}**H NMR** (400 MHz, DMSO-d_6) δ 8.03 (1H, s, H2), 7.07 (1H, s, H8), 6.54 (2H, br s, 6-NH_2), 6.04 (1H, d, J = 7.4 Hz, H1′), 5.55 (2H, br s, α-NH_2), 4.40 (1H, t, J = 5.6 Hz, H2′), 4.03 (1H, dd, J = 5.6, 3.9 Hz, H3′), 3.94 (1H, q, J = 6.1 Hz, H4′), 3.30 (1H, dd, , J = 7.5, 5.6 Hz, Hα), 2.87 (1H, dd, J = 13.6, 6.0 Hz, H5′a), 2.78 (2H, q, J = 7.3 Hz, CH_3**CH_2**), 2.72 (1H, dd , J = 13.9, 6.7 Hz, H5′b), 2.63 (2H, t, , J = 7.7 Hz, Hγ), 2.07−1.94 (1H, m, Hβa), 1.90−1.76 (1H, m, Hβb), 1.20 (3H, t, J = 7.4 Hz, **CH_3**CH_2). ^{13}**C NMR** (101 MHz, DMSO-d_6) δ 169.9 (**CO**OH), 157.8 (C6), 151.7 (C2), 151.4 (C4), 118.0 (C8), 117.6 (C7), 102.2 (C5), 86.5 (C1′), 82.7 (C4′), 73.2 (C2′), 72.8 (C3′), 53.2 (Cα), 34.3 (C5′), 31.6 (Cβ), 28.4 (Cγ), 19.4 (CH_3**CH_2**), 15.1 (**CH_3**CH_2). **HRMS** (ESI) $[M + H]^+$ m/z calcd. for $C_{17}H_{26}O_5N_5S$, 412.1649; found, 412.1649.

NMR spectra - To validate the purity and chemical structure of the new compounds, an NMR ^{1}H and ^{13}C NMR spectra was obtained for each of the new compounds. The spectra clearly illustrate high purity co the prepared compounds (SI Figure 4 - 8).

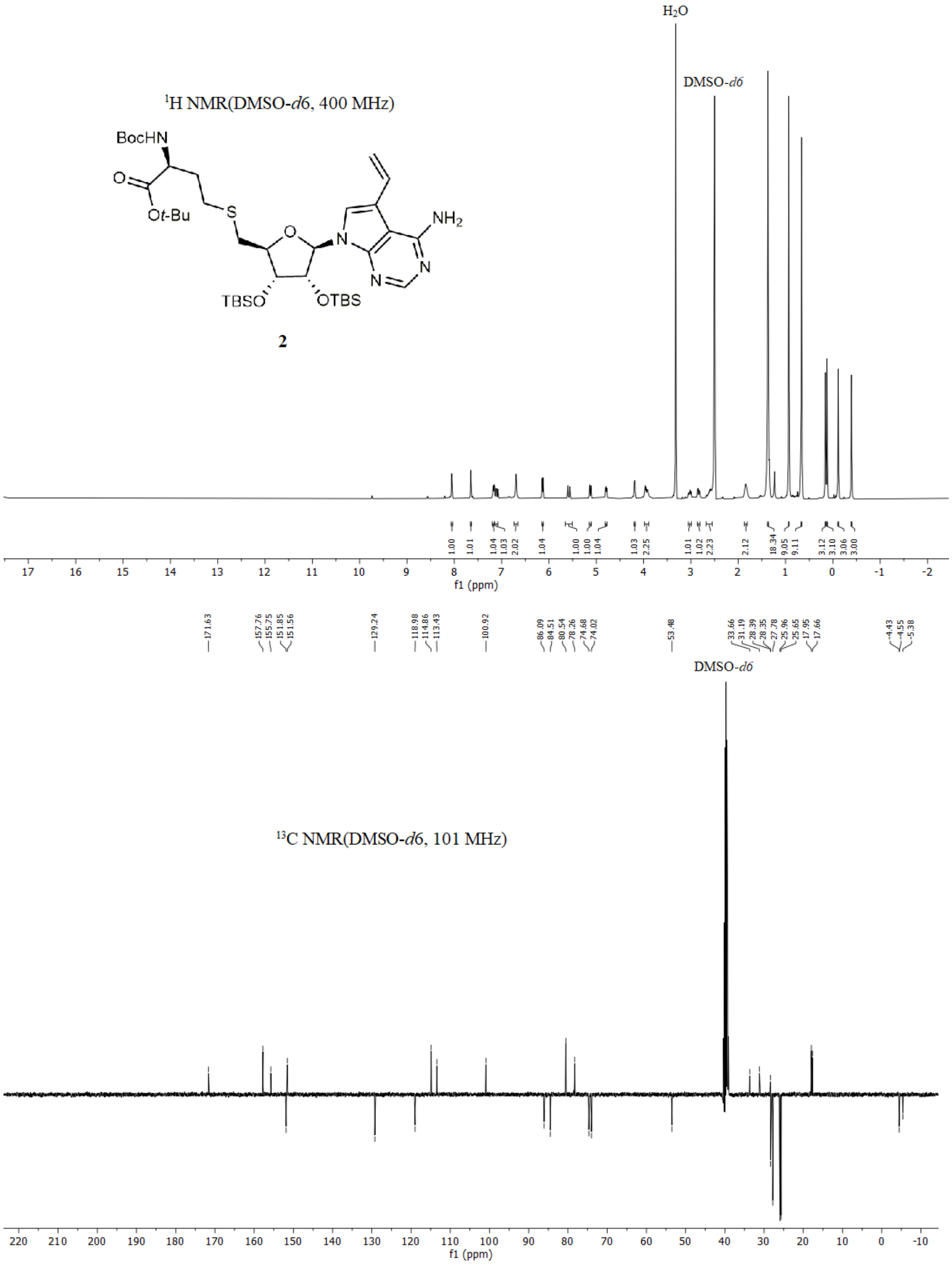

Supplementary Figure 4. ^{1}H (top) and ^{13}C APT (bottom) NMR spectra of compound **2** measured in DMSO-*d6* at r.t. (25 °C).

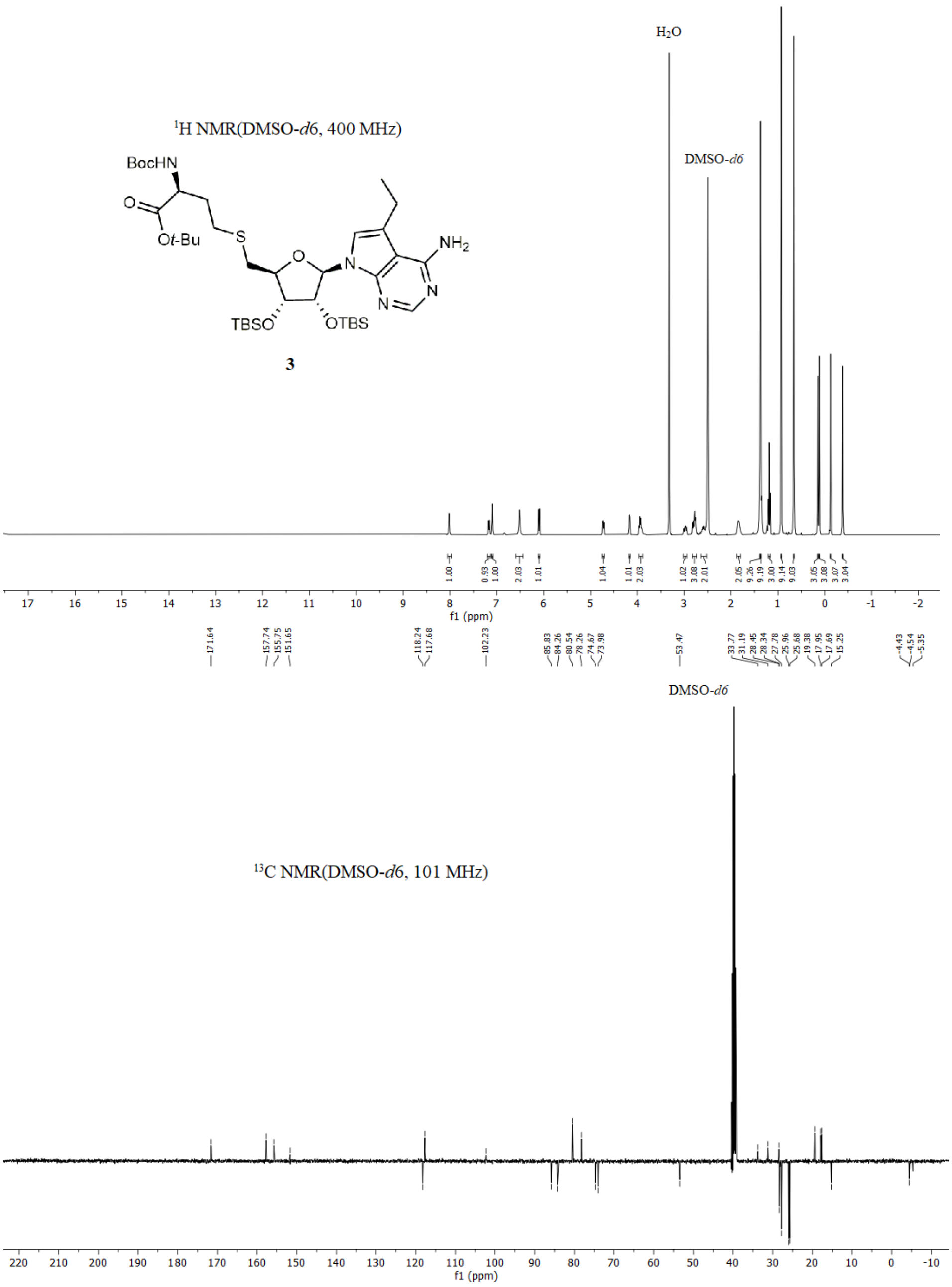

Supplementary Figure 5. ^{1}H (top) and ^{13}C APT (bottom) NMR spectra of compound **3** measured in DMSO-*d6* at r.t. (25 °C).

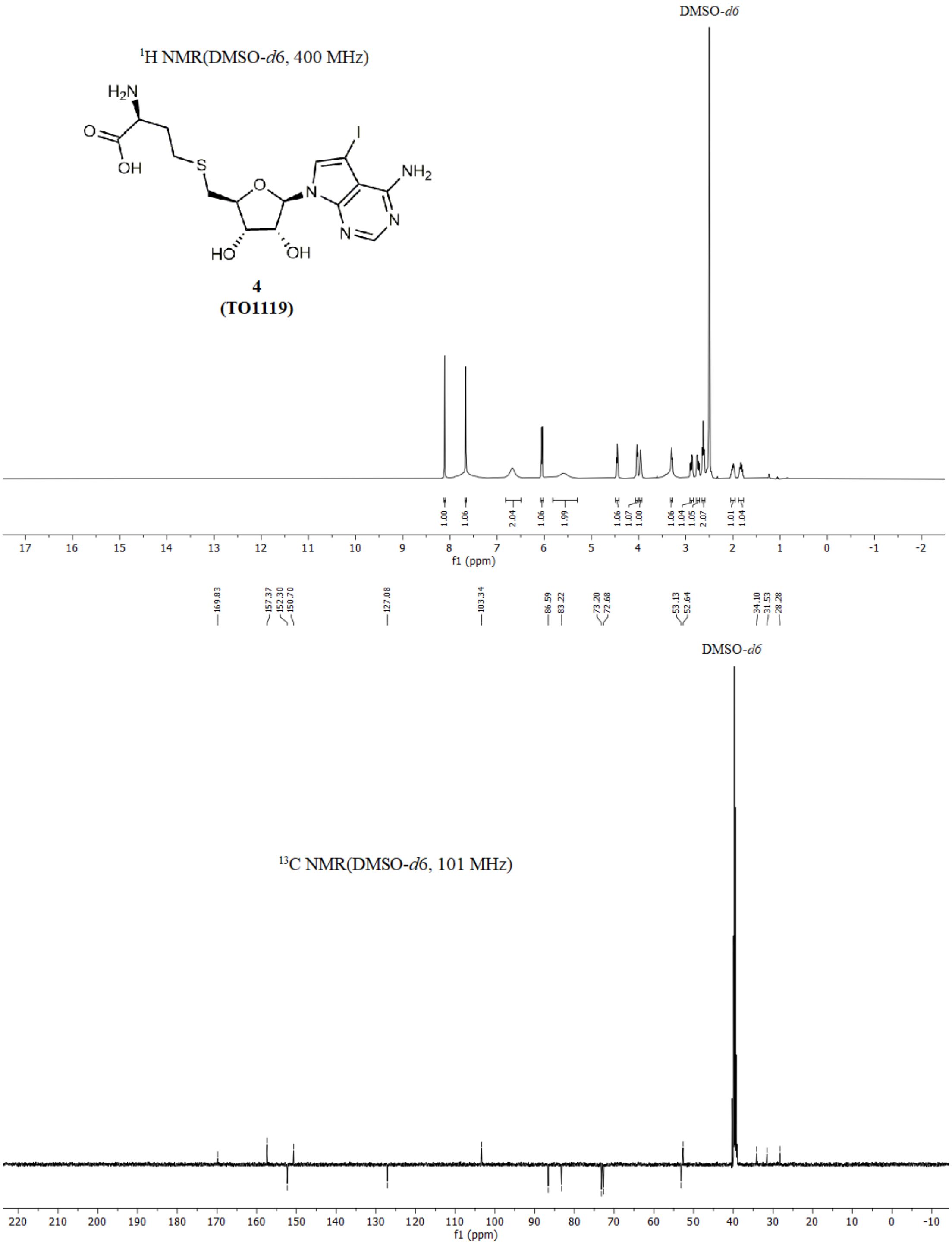

Supplementary Figure 6. ^{1}H (top) and ^{13}C APT (bottom) NMR spectra of compound **4 (TO1119)** measured in DMSO-*d6* at r.t. (25 °C).

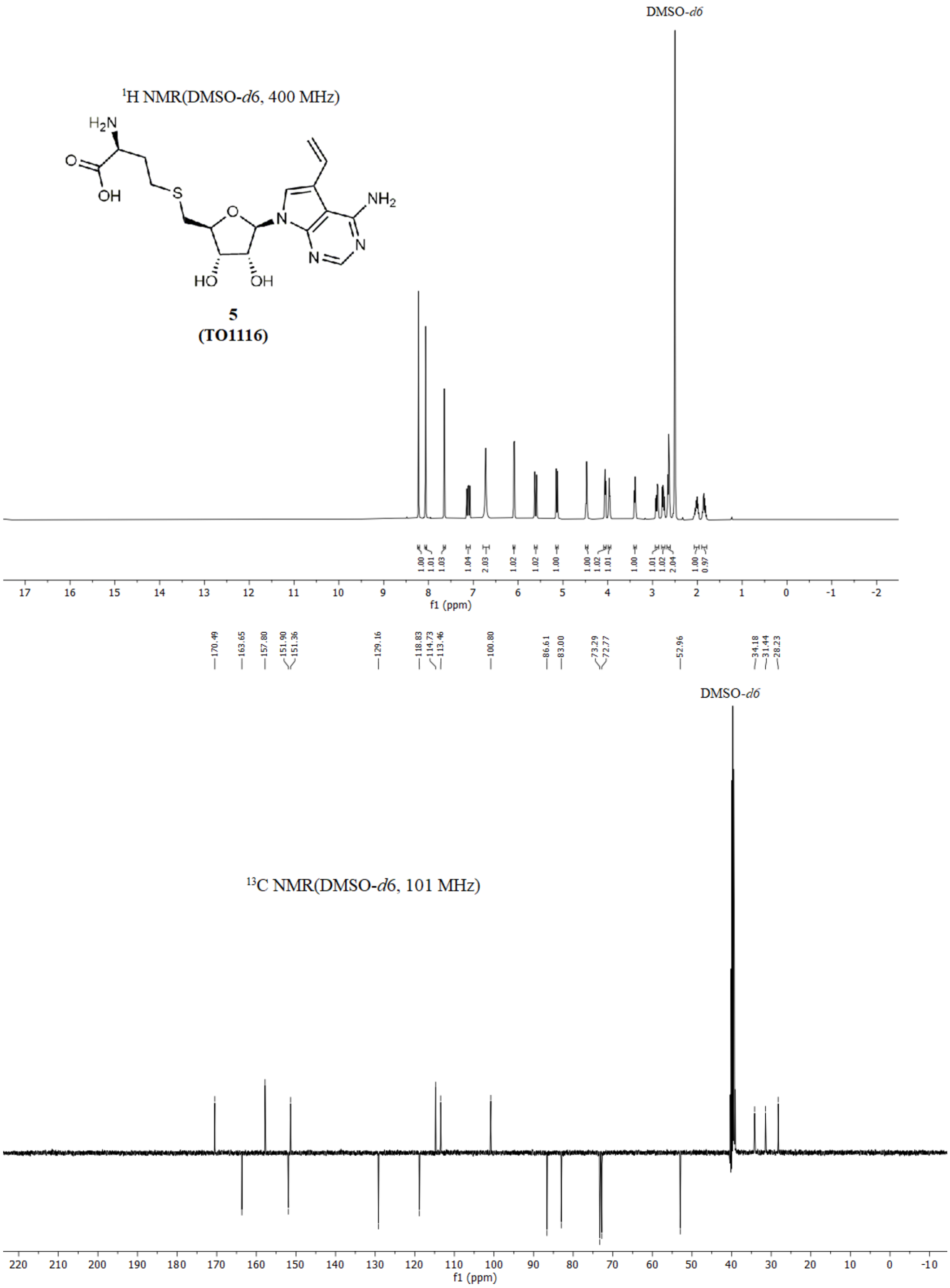

Supplementary Figure 7. ^{1}H (top) and ^{13}C APT (bottom) NMR spectra of compound **5 (TO1116)** measured in DMSO-*d6* at r.t. (25 °C).

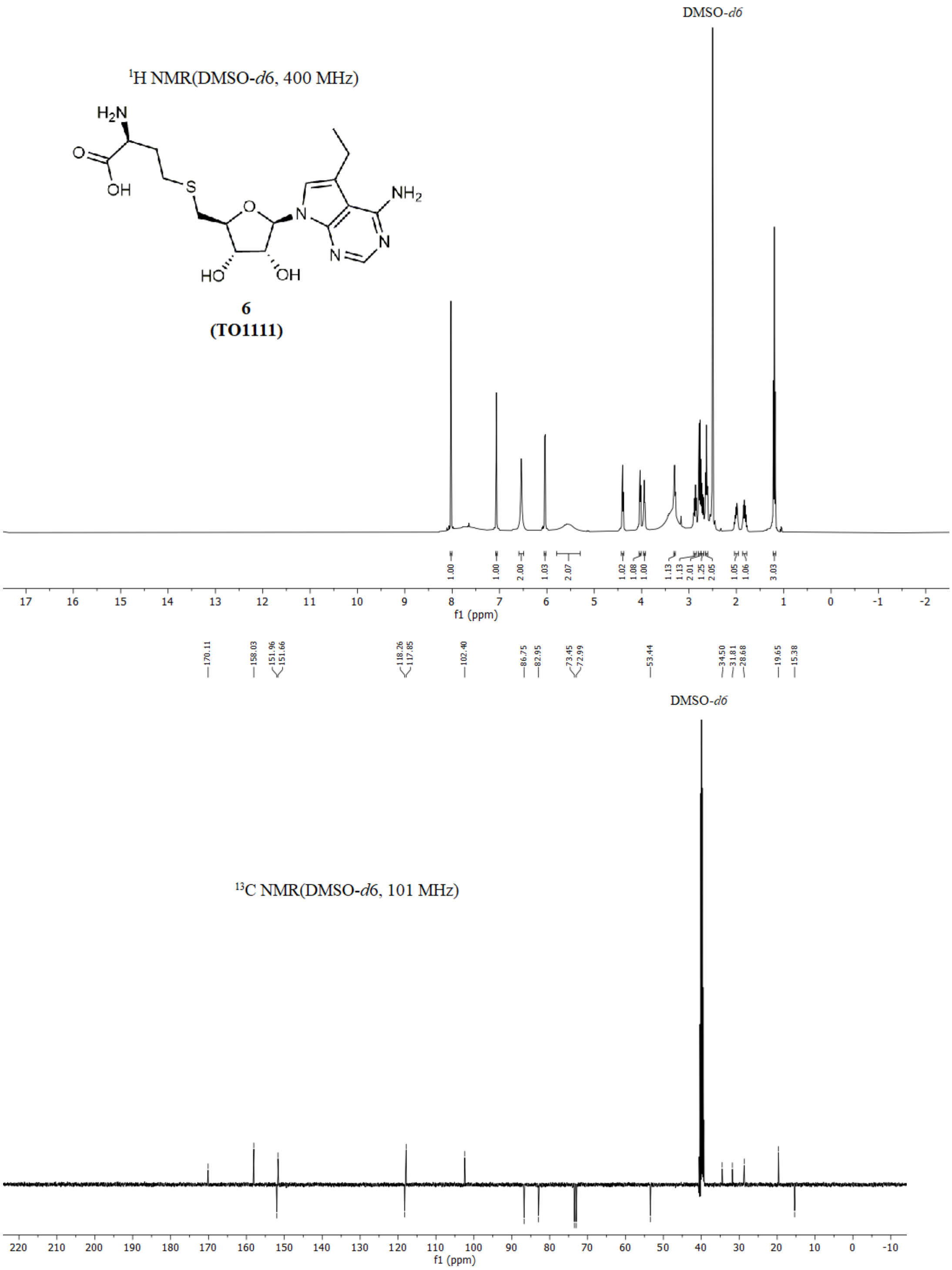

Supplementary Figure 8. ^{1}H (top) and ^{13}C APT (bottom) NMR spectra of compound **6 (TO1111)** measured in DMSO-*d6* at r.t. (25 °C).

Supplementary References

1. Otava, T.; Šála, M.; Li, F.; Fanfrlík, J.; Devkota, K.; Perveen, S.; Chau, I.; Pakarian, P.; Hobza, P.; Vedadi, M.; Boura, E. and Nencka, R. (2021) The Structure-Based Design of SARS-CoV-2 nsp14 Methyltransferase Ligands Yields Nanomolar Inhibitors. *ACS Infect. Dis. 7 (8)*, 2214–2220.
